# Causes of mortality in elderly UICC stage III colon cancer (CC) patients––Tumor‐related death and competing risks from the German AIO colorectal study group Colopredict Plus (CPP) registry

**DOI:** 10.1002/cam4.4540

**Published:** 2022-02-11

**Authors:** Stefanie Nöpel‐Dünnebacke, Hendrick Jütte, Robin Denz, Inke Sabine Feder, Anna‐Lena Kraeft, Celine Lugnier, Christian Teschendorf, Daniela Collette, Hinrich Böhner, Lars Engel, Lothar Mueller, Frank Hartmann, Ulrich Kaiser, Harald‐Robert Bruch, Stephan Hollerbach, Dirk Arnold, Nina Timmesfeld, Andrea Tannapfel, Anke Reinacher‐Schick

**Affiliations:** ^1^ Department of Hematology, Oncology and Palliative Care St. Josef‐Hospital Bochum Ruhr‐University Bochum Germany; ^2^ Institute of Pathology Ruhr‐University Bochum Bochum Germany; ^3^ Department of Medical Informatics Biometrics and Epidemiology Ruhr‐University Bochum Germany; ^4^ Department of Internal Medicine St. Josefs Hospital Dortmund Dortmund Germany; ^5^ Joint Practice of Hematology and Oncology Catholic Hospital Dortmund West Dortmund Germany; ^6^ Department of General and Visceral Surgery Catholic Hospital Dortmund West Germany; ^7^ Department of General and Visceral Surgery Paracelsus Medical University Hospital Nürnberg Nord Nürnberg Germany; ^8^ Onkologie UnterEms Leer Germany; ^9^ Department of Oncology and Hematology Klinikum Lippe Lemgo Germany; ^10^ Department of Hematology, Oncology and Palliative Care St. Bernsward Hospital Hildesheim Germany; ^11^ Joint Practice of Oncology and Hematology Bonn Germany; ^12^ Department of Gastroenterology AKH Celle Celle Germany; ^13^ Department of Oncology, Hematology and Palliative Care Asklepios Hospital, Cancer Center Altona Hamburg Germany

**Keywords:** adjuvant treatment, early colon cancer, elderly, tumor‐related death

## Abstract

**Background:**

Colon cancer (CC) is a disease of elderly patients (pts.) with a median age of 73 years (yrs.). Lack of data about the effects of adjuvant chemotherapy (ACT) is caused by underrepresentation of this clinically relevant cohort in interventional trials. We analyzed real‐world data from the German CPP registry with regard to a possible benefit of ACT in elderly (70+ yrs.) versus younger pts. (50 to <70 yrs.) taking cause‐specific deaths into account.

**Methods:**

We analyzed the effect of age and ACT on overall survival (OS) and cause‐specific death of stage III pts. using Cox regression.

**Results:**

In total, 1558 pts. were analyzed and follow‐up was 24.6 months. 62.6% of the elderly received ACT whereas 91.1% of younger pts. (*p *< 0.001). Oxaliplatin combinations were significantly less often given to older than younger pts. (38.8% vs. 88.9%; *p *< 0.001). Mean Charlson comorbidity score was significantly lower in pts. that received ACT (0.61) than in those without ACT (1.16; *p *< 0.001). ACT was an independent positive prognostic factor for cancer‐related death in elderly pts. even in pts. 75+ yrs. No significant difference in the effect of ACT could be observed between age groups (interaction: cancer‐specific death HR = 1.7948, *p *= 0.1079; death of other cause HR = 0.7384, *p *= 0.6705).

**Conclusion:**

ACT was an independent positive prognostic factor for OS. There may be a cohort of elderly with less co‐morbidities who benefit from ACT.

## Graphical abstract

More than half of CC cases are diagnosed in patients aged over 70 years who form a particularly heterogeneous cohort regarding functional status, comorbidities, and social support. Guidance in cancer‐treatment decisions is complicated by a lack of high‐quality evidence. Our analysis evaluated the impact of ACT on survival and causes of death in elderly with CC within the real‐world AIO registry CPP. ACT was an independent positive prognostic factor for cancer‐related death; OS and DFS were significantly higher in pts. who received ACT. These data implicate that elderly with few comorbidities eligible to receive ACT, might benefit like their younger counterparts

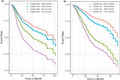

## 1 INTRODUCTION

Colon cancer (CC) still ranks among the most frequent types of malignant neoplasms and still accounts annually for almost 30,000 deaths in Germany.^1^ With a median age of 73 years (yrs.) CC is a disease of elderly.^2^ Numerous randomized trials (RCTs) have proven the benefit of adjuvant chemotherapy (ACT) regarding survival in UICC stage III pts.^3^, ^5^ The median age of included patients (pts.), however often undercuts the statistical median age of the disease. Hence, elderly pts. are regularly underrepresented in RCTs and less likely to be offered systemic treatment.^6^, ^8^ Therefore, robust data on the benefit of ACT in elderly CC pts. are scarce and national and international guideline recommendations are controversial.^9^, ^10^ The TOSCA trial (part of the IDEA collaboration that investigated the non‐inferiority of 3 vs. 6 months ACT), published data concerning pts. >70 yrs. and showed no differences in pts. below or above 70 yrs. and duration of treatment (HR 1.15 for both^11^, ^12^). Data from a pooled analysis of >70 yrs. stage II/III CC pts. not only indicated a benefit on survival but also showed similar rates of adverse events.^13^, ^14^, ^15^, ^16^ Several tools are established to determine the life expectancy in older cancer pts., for example, the Charlson comorbidity score. Charlson comorbidity score scores age, co‐morbidities, and their severeness in cancer pts., where Charlson comorbidity score >5 correlates with a shorter OS.^17^

We aimed to analyze whether older pts. at or above 70 yrs. compared to younger (50 to <70 yrs.) have a similar benefit from ACT in UICC stage III CC, particularly with regard to cause‐specific death.

## 2 MATERIALS AND METHODS

In September 2013, the molecular registry trial CPP was initiated in 70 German community cancer centers (Blinded for peer review). Newly diagnosed, histopathologically confirmed UICC stage II or III CC pts. were included after surgery and written informed consent. Of note, UICC stage I and IV as well as rectal cancer were excluded. Data cutoff for this subgroup analysis of elderly (≥50 yrs.) UICC stage III pts. with a Charlson comorbidity score <5 was October 29, 2020. Cause of death, as stored in the database, was monitored by a GI oncologist for plausibility according to Raycraft et al.^18^ A no traceable cause of death was set to “unknown.”

### 2.1 Patients

For all included CC pts., the following parameters were assessed: gender, age, weight, height, body mass index (BMI), and acquired co‐morbidities. These parameters were used to calculate Charlson comorbidity score, UICC stage, primary tumor localization, adjuvant treatment regimen, respectively, applied medication (oxaliplatin and fluoropyrimidine), clinical and histopathological risk factors, secondary malignancies, molecular markers (BRAF and RAS mutational status, microsatellite status), disease‐free survival (DFS), and overall survival (OS). In detail, Charlson comorbidity score was calculated using co‐morbidities such as secondary malignancies (breast, lung, gynecologic, prostate, and others), pulmonary or cardiovascular diseases, organ dysfunctions (gastrointestinal, renal, and liver) plus metabolic diseases like rheumatologic disorders, HIV, and diabetes mellitus analogous to Colinet et al.^17^ To reveal potential differences between younger and older CC pts., we established two age groups 50 to <70 yrs. versus 70+ yrs. Because German Guidelines do not recommend ACT for patients aged 75+ we also analyzed a subgroup of 599 pts. 75+ yrs.

### 2.2 Histology and molecular analysis

Molecular analysis was performed centrally by Institute of Pathology via next‐generation sequencing (NGS) molecular markers like BRAF and RAS (K‐ and N‐RAS) mutational status were assessed. Tumor DNA was amplified with a custom Primer Panel (Qiagen, GeneRead V2), ligated to adaptors (BIOO Scientific) and analyzed on a MiSeq Sequencer (Illumina). A mutation was considered as valid with a mutation frequency of at least 5%, read balance >0.1, and coverage >400. A substantial number of samples has been analyzed using the Human Colorectal Cancer Panel (Qiagen). Isolated tumor DNA is coded with unique molecular identifiers (QIAseq 96‐Index I Set C [384]) and subsequently amplified and analyzed on the NextSeq 550 (Illumina). A mutation was considered as valid with at least 5% allelic frequency, read balance >0.3, and coverage >100. Microsatellite analysis was performed via immunohistochemistry of MMR proteins and fragment length analysis analogous to Boland et al.^19^

### 2.3 Statistics

Arithmetic means and standard deviations were calculated for continuous variables, frequencies and percentages for categorical variables. Differences between age groups were tested with *t*‐tests and chi‐squared tests. OS was defined with death of any cause (regardless of relapse). All pts. lost to follow‐up (defined as time to last follow‐up >6 months) and alive pts. were censored. DFS combined both endpoints relapse and death. Confounder‐adjusted survival curves were calculated using direct standardization based on cox regression models. Cause‐specific cumulative incidence functions considering competing risks were estimated using a nonparametric Aalen–Johansen estimator. Hazard ratios (HRs) with 95% confidence intervals (CIs) were calculated using a cause‐specific cox regression model. *P* values were defined as significant, when *p *< 0.05.

## 3 RESULTS

### 3.1 Patients

**FIGURE 1:**
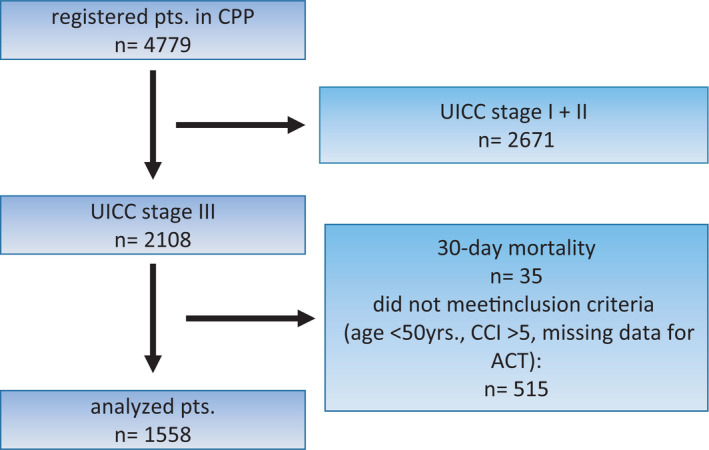
Consort diagram of the subgroup analysis of CPP

In total, 4779 pts. were registered within CPP until data cutoff on October 29, 2020, of those 2108 had UICC stage III CC. For this subgroup analysis we excluded pts. with a Charlson comorbidity score ≥5, pts. <50 yrs., patients who had not survived surgery (mortality within 30 days after surgery), and patients with missing data for adjuvant treatment. In summary, 515 pts. had to be excluded from the final evaluation (Figure 1). Median follow‐up was 24.6 months. A total of 1558 pts. were included in this subgroup analysis comprising 745 (48%) female and 808 (52%) male subjects. The group of pts. ranging from 50 to <70 yrs. included 688 (44.2%) pts., while 870 pts. were 70+ yrs. (55.8%). The median Charlson comorbidity score was significantly lower in the younger age group 0.52 (50 to <70 yrs.) versus 0.92 (70+ yrs.); *p *< 0.001 (Table 1). Pts. who received ACT had a median Charlson comorbidity score of 0.61, whereas the untreated cohort displayed a mean CC of 1.16 (*p *< 0.001). CCs with a primary tumor localization in the right colon were more frequent in the whole study population (58.6%) as well as in the elderly (63.3% vs. 52.6%; *p *< 0.001; Table 1). In 42 pts. (2.7%), the localization was overlapping or not precise.

**TABLE 1:** Baseline characteristics according to age groups of UICC stage III patient enrolled into the CPP registry

	Total	50 to <70 yrs.	70+ yrs.	*p* value	75+ yrs.
*N* (%)	1558	688 (44.2%)	870 (55.8%)		599 (38.4%)
Female	745 (48.0%)	297 (43.2%)	448 (51.7%)		313 (52.5%)
Male	808 (52.0%)	390 (56.8%)	418 (48.3%)	0.001	283 (47.5%)
CCS (median)	0.75	0.52	0.92	<0.001	0.993
Localization					
Left	627 (41.4%)	314 (47.4%)	313 (36.7%)		209 (34.9%)
Right	889 (58.6%)	349 (52.6%)	540 (63.3%)	<0.001	382 (63.8%)
Missing	42 (2,7%)				7 (1.2%)
ACT					
No	386 (24.8%)	61 (8.9%)	325 (37.4%)		279 (46.6%)
Yes	1172 (75.2%)	627 (91.1%)	545 (62.6%)	<0.001	320 (53.4%)
FP monotherapy	390 (34.2%)	68 (11.1%)	322 (60.9%)		229 (71.6%)
Ox‐containing	747 (65.6%)	542 (88.9%)	205 (38.8%)	<0.001	79 (24.7%)
Missing	35 (3%)				12 (3.7%)

Abbreviations: ACT, adjuvant chemotherapy; CCS, Charlson co‐morbidity score; FP, fluoropyrimidine; Ox, oxaliplatin; yrs., years.

### 3.2 Adjuvant chemotherapy

Out of 1558 included pts. 1172 (75.2%) pts. received ACT: 627 (91.1%) pts. comprised the age group 50 to <70 yrs., whereas 545 (62.6%) pts. were 70 yrs. and older (*p *< 0.001, Table 1). In total, 386 pts. did not receive ACT: 61 (8.87%) in the younger and 325 (37.4%) from the older pts. group. Fluoropyrimidine monotherapy was given to 390 (34.2%) pts., 68 (11.1%) in the younger cohort and 322 (60.9%) in the older population. A total of 747 (65.6%) pts. received oxaliplatin‐containing ACT, 542 (88.9%) in the 50 to <70 yrs. cohort and 205 (38.8%) of ≥70 yrs. of age (*p *< 0.001, Table 1). In 35 cases (3%) data were missing. Furthermore, we analyzed a subgroup of the 70+ yrs. cohort. This were 599 pts. including and above 75 yrs. concerning ACT and regimen. In total, 320 pts. (53.4%) received ACT thereof 229 pts. (71.6%) received fluoropyrimidine monotherapy and 79 pts. (24.7%) received oxaliplatin‐containing regimen.

### 3.3 Mutational analysis

**FIGURE 2:**
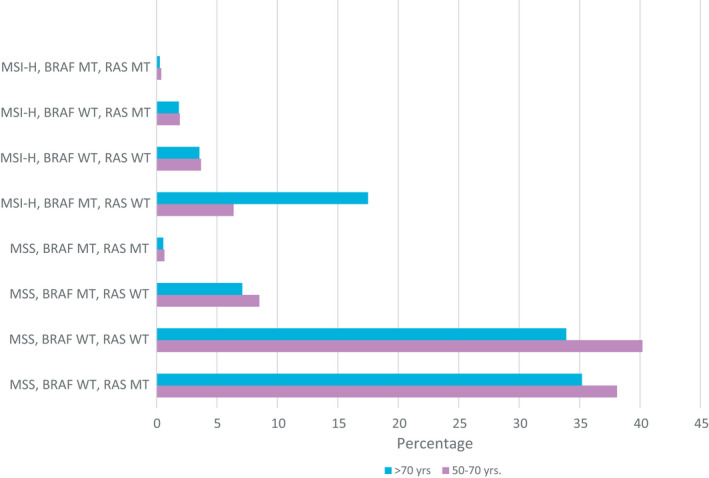
Barplot of the frequency of molecular alterations such as RAS/BARF MT and MSI‐H versus MSS. MSI‐H, high microsatellite instability; MSS, microsatellite stability; MT, mutation; WT, wildtype

Microsatellite status (microsatellite instable [MSI‐H]) vs. stable (MSS), BRAF‐ and RAS‐mutational status were analyzed. Among the age group ≥70 yrs. a higher percentage BRA‐mutated and MSI‐H tumors occurred, while younger patients had more MSS and BRAF wild‐type tumors (Figure 2).

### 3.4 Survival

**FIGURE 3:**
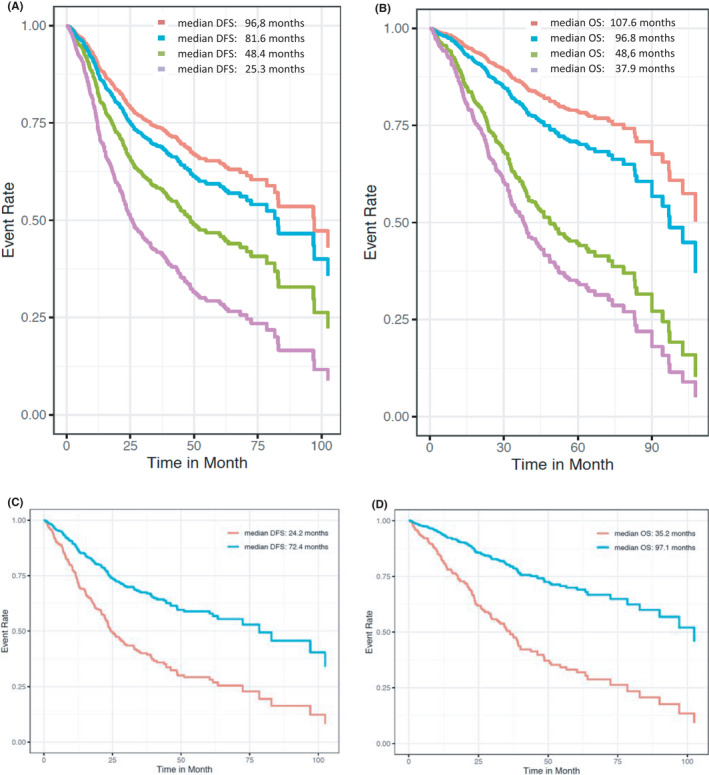
**(**A and B) median disease free survival (DFS, A) and overall survival (OS, B) in UICC stage III patients (pts.) in age groups (50 to <70 vs. 70+ yrs.) and with vs. without adjuvant chemotherapy (ACT) adjusted to the Charlson Comorbidity Score (CCS); red: pts. 50 to <70 yrs. with ACT; blue: pts. 70+ yrs. with ACT; green: pts. 50 to <70 yrs. without ACT; violet: pts. 70+ yrs. without ACT. (C and D) disease free survival (DFS, C) and overall survival (OS, D) in UICC stage III patients (pts.) 75+ yrs. with vs. without adjuvant chemotherapy (ACT) adjusted to the Charlson Comorbidity Score (CCS); blue: with ACT, red: without ACT

Survival data for DFS (*n* = 1348) and OS (*n* = 1558) were available. For both survival analysis endpoints, the following four subgroups were considered: pts. with versus without ACT in relation to both age groups 50 to <70 yrs. versus 70+ yrs. These analyses were adjusted by Charlson comorbidity score (Figure 3A,B). Concerning DFS and OS, both age groups had an improved survival when receiving ACT. Median DFS of treated pts. 50 to <70 yrs. was 96.8 versus 81.6 months in pts. 70+ yrs. compared to 48.4 and 25.3 months without ACT. Similar results were obtained in OS: median OS in the younger group was 107.6 and 96.8 months in the older age group with ACT versus 48.6 and 37.9 months without ACT (Figure 3A,B). Additionally, 3‐ and 5‐yr. DFS and OS were calculated in relation to both age and ACT, details are shown in Table 2. To assess the effect of ACT in pts. including and above the age of 75 yrs., we also performed DFS and OS analysis in this subgroup. CCS adjusted survival curves were calculated split by ACT status (Figure 3C,D).

**TABLE 2:** Three and 5 yrs. DFS and OS in relation to age groups and ACT

	3 yrs. DFS (%)	3 yrs. OS (%)	5 yrs. DFS (%)	5 yrs. OS (%)
ACT				
50 to <70 yrs.	73.9	86.5	64.9	78.6
70+ yrs.	69.1	81.1	59.0	70.5
No ACT				
50 to <70 yrs.	58.3	61.5	46.3	44.6
70+ yrs.	41.8	52.7	28.9	34.6

Abbreviations: ACT, adjuvant chemotherapy; DFS, disease free survial; OS, overall survival; yrs, years.

### 3.5 Cause‐specific death

**FIGURE 4:**
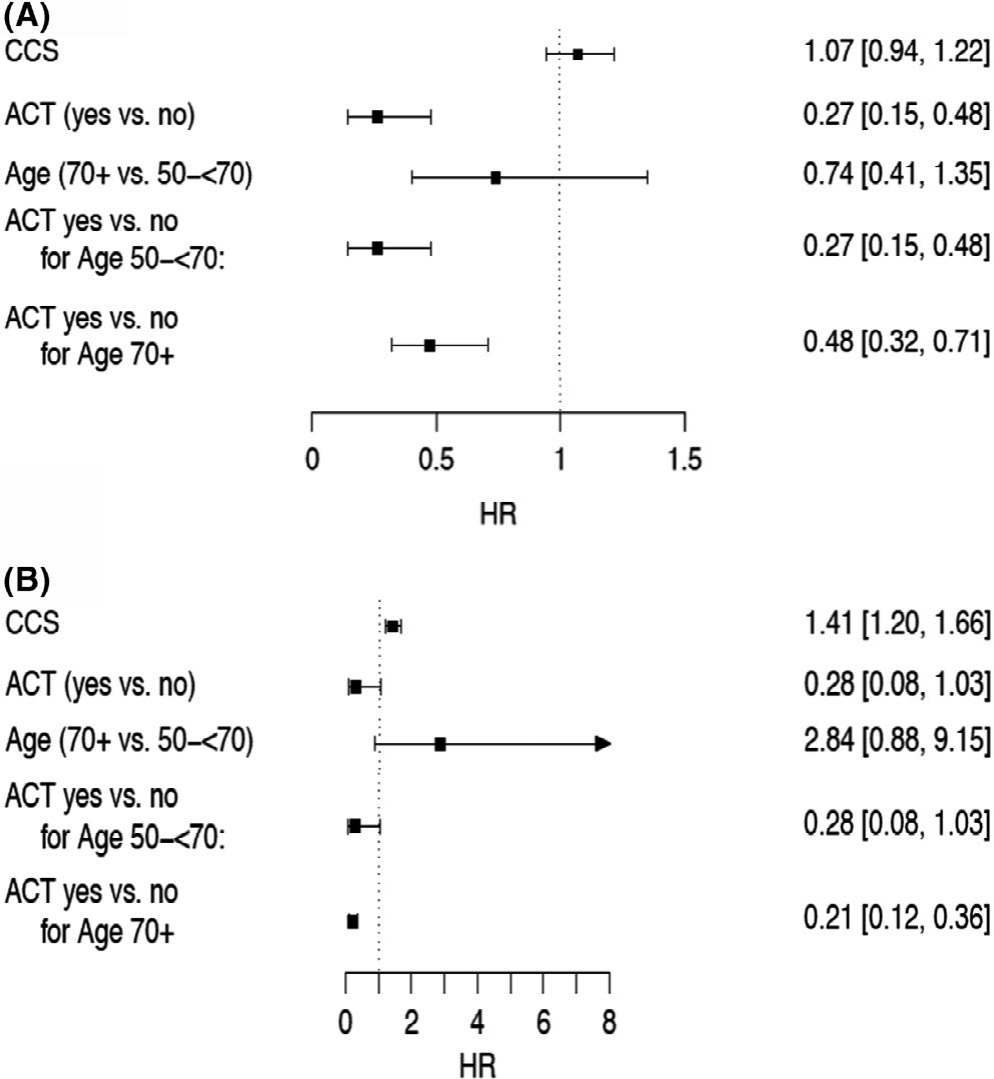
Forest plots for cancer specific death (A) and death from other causes (B); Hazard ratio (95% Confidence interval)

**FIGURE 5:**
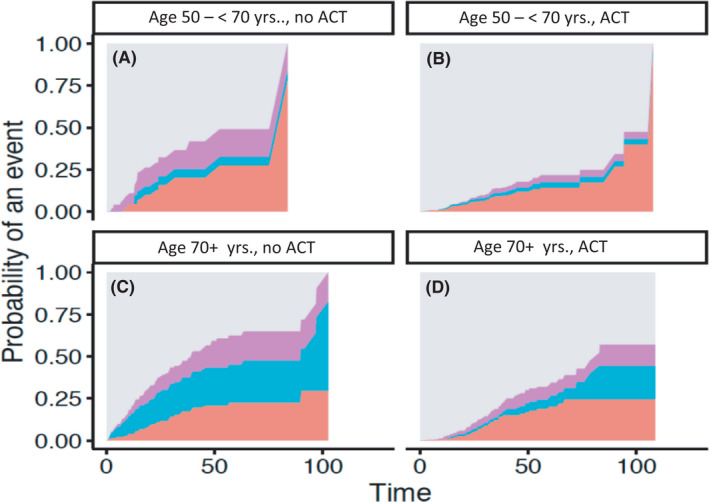
Causes of death od UICC stage III pts. in two age groups (50 to <70 yrs. (A, B). vs. 70+ yrs. (C, D)) and adjuvant chemotherapy (ACT; B, D) and no treatment (A, C). Grey: alive pts., violet: unknown death, blue: other cause, red: cancer

In addition to standard survival analysis, we performed analyses discriminating between cancer‐related deaths, deaths of other causes, and deaths of unknown cause. A total of 268 pts. (17.2%) died during the time of follow‐up. In total, 131 out of 268 (48.9%) pts. died cancer related, 44 out of 64 (68.8%) in the younger ACT group, 9 out of 17 (52.9%) from the younger age group without ATC, 44 out of 79 (55.7%) from the older age group with ACT, and 34 out of 108 (31.5%) without treatment. Five chemotherapy‐associated deaths occurred and were counted as cancer related. Seventy‐three out of 268 pts. deaths were not cancer related. Cancer‐independent deaths were subdivided into metabolic, cardiovascular, neurological diseases, and infection. The most frequent deaths were cardiovascular‐related deaths especially in pts. 70+ yrs. In summary, elderly died more often due to other causes (13.6% vs. 33.2%). In 64 events, causes of death were unknown. In 158 events a secondary malignancy was documented: 56 (8.2%) in the younger group and 102 (11.8%) in the older group (*p *= 0.005). Using a cause‐specific Cox regression approach, we performed a multivariate analysis adjusting for Charlson comorbidity score while including the two age groups and ACT as interaction term. Figure 5A–D shows the cause‐specific cumulative incidence functions according to age groups and ACT treatment. Pts. in both age groups treated with ACT had a lower risk for cancer‐specific death (50 to <70 yrs.: HR = 0.27; 95% CI 0.15–0.48, *p *< 0.001 and 70+ yrs.: HR = 0.48; 95% CI 0.32–0.71; *p *< 0.001, Figures 4A and 5B). Elderly receiving ACT were also less likely to die from other causes (70+ yrs.: HR = 0.21; 95% CI 0.12–0.36, *p *< 0.001), which was observed in their younger counterparts, but did not reach defined significance level (50 to <70 yrs.: HR = 0.28; 95% CI 0.08–1.03, *p *= 0.05; Figures 4B and 5D). A higher Charlson comorbidity score correlated with cancer‐related death (HR 1.07; 95% CI 0.94–1.22, *p *= 0.27; Figure 4A), yet did not reach the defined significance threshold of *p *< 0.05. In non‐cancer‐related deaths, however, the effect of a higher Charlson comorbidity score on survival was statistically significant (HR 1.41; 95% CI 1.20–1.66, *p *< 0.001; Figure 4B). There was no significant difference in the effect of ACT between age groups concerning cause of death (interaction: cancer‐specific HR = 1.7948, *p *= 0.1079; other cause HR = 0.7384, *p *= 0.6705). Due to convergence issues caused by low sample sizes when estimating the cause‐specific Cox model, we cannot report effect estimates for unknown causes of death.

## 4 DISCUSSION

This analysis of a subgroup of a fit (Charlson comorbidity score <5) elderly cohort (≥50 yrs.) of the German AIO molecular registry trial CPP evaluated the use of ACT and causes of death in UICC stage III CC pts. in two age groups (50 to <70 yrs. vs. 70+ yrs.). Elderly and explicit pts. including and above the age of 75 yrs. seem to benefit from ACT similar to younger pts. ACT was an independent positive prognostic factor for survival. In both age groups pts. treated with ACT had a lower risk to die from cancer‐specific causes and to die from other causes. A higher Charlson comorbidity score correlated with shorter survival especially for death from other causes.

### 4.1 Clinical and molecular parameters

In accordance with previous published data our cohort consisted of an elderly population with a median age of 73 yrs. Our cohort contained slightly more men than women (52% vs. 48%) and right‐sided CCs.^20^, ^21^ Among the elderly, BRAF‐mutated MSI‐H tumors were more frequent.^21^, ^22^, ^23^ As anticipated, co‐morbidities calculated with the Charlson comorbidity score were significantly higher in elderly than in younger pts. Mean Charlson comorbidity score of pts. who did not receive ACT was 1.16 compared to the cohort that received treatment 0.61 (*p *< 0.001).

### 4.2 Adjuvant treatment

In our analysis 75.2% of the whole cohort received ACT, respectively, 91.1% of the younger and 62.2% of the elderly pts. To eliminate the impact of age‐dependent co‐morbidities and within treatment recommendations, CCS was calculated for each pt. and pts. with a CCS ≥5 were excluded. Still treatment significantly differed between both age groups. Elderly patients were significantly less frequently assigned to treatment and, when receiving ACT, were more likely to receive fluoropyrimidine monotherapy.^24^ However, despite the lack of evidence and guideline recommendations, oxaliplatin combinations were applied in 38.8% of pts.^9^ Surprisingly 53.4% of pts. including and above the age of 75 yrs. received ACT, even 24.7% oxaliplatin‐containing protocols, which may represent an older fitter population, who benefit from ACT and which need to be detected. Other analyses of registry data with focus on UICC III CC pts. that explored intensity of treatment, toxicity, and guidance among elderly state similar results to our findings.^13^, ^18^, ^24^, ^25^ The proportion of pts. receiving ACT ranged from 81% to 91% among younger versus 48%–60% in elderly. Elderly were less frequently assigned to ACT, which could not be exclusively explained by higher comorbidity scores.^25^ The treatment drop‐out rate (due to side effects, progression, patient´s choice, etc.) was equally distributed between the elderly and their younger counterparts indicating that age was the most decisive aspect in regard to ACT.^13^ Data regarding the issue whether oxaliplatin for ACT is beneficial and can be safely administered in elderly as well, are sparse but there are indications reported in the literature. Haller et al. showed a benefit in all age groups (<70 yrs. vs. >70 yrs.) the adjuvant treatment of UICC stage III CCs via a pooled analysis of four RCTs (NSABP C‐08, XELOXA, X‐ACT, and AVANT), indicating an improved DFS and OS regardless of age and co‐morbidities (<70 yrs.: HR 0.68; >70 yrs. HR 0.77), while displaying a comparable rate of serious adverse events.^9^, ^10^, ^26^ In contrast to these results stand the ACCENT database analysis. Pooled data from three studies (MOSAIC, NSABP‐07, and XELOXA) suggested limited benefit from adding oxaliplatin in elderly pts. (HR for OS 1.04^27^).

### 4.3 Survival and cancer versus non‐cancer‐related deaths

ACT was an independent prognostic factor for DFS and OS in both age groups, which is consistent to published data.^24^ ACT clearly improved the 3‐ and 5‐yr. DFS and OS (Table 2), even in pts. aged 75 yrs. or older. Additionally, pts. who received ACT had a lower risk for cancer‐related death (coherent in both age groups) and, especially in elderly, for non‐cancer‐related death. Death from other causes did correlate with the registered co‐morbidities. A higher Charlson comorbidity score (in both age groups) did also correlate with shorter survival, emphasizing that geriatric assessment might be useful to set disabilities versus age into a balanced perspective when considering pt. eligibility for ACT. Recommendations for ACT in UICC stage III pts. evolved from large RCTs, thereby establishing recommendations in clinical routine that are based on data from a significantly younger cohort.^3^, ^4^

The elderly are frequently not represented adequately in RCTs, which is often due to the objectives and study concept in prospective RCTs, resulting in low evidence regarding recommendations for treatment.^7^, ^8^, ^9^, ^28^, ^29^, ^30^ Data referring to ACT is mostly generated within retrospective analyses or registries and hence maybe less recognized, although these data hint feasibility and benefit of ACT.^18^, ^26^ There is strong consensus on the cautious use of ACT in elderly patients. It must be assessed and discussed, though, whether eligible pts. should receive standard therapy, without making age the main criterion for decision on ACT. In general, pts. with a life expectancy <5 yrs. should not be offered ACT, while those with a longer life expectancy should receive standard therapy.^9^, ^18^ To identify pts. at risk for toxicity and death of other causes as well as pts. eligible for intensive treatment, geriatric assessment (GA) should be standard of care and hence be performed in all cases. A GA is helpful to balance (severe) co‐morbidities, fragility, and mental impairments^7^, ^8^, ^31^ regarding a well‐founded decision on ACT.

## 5 SUMMARY

In summary, our results suggest that elderly pts. 70+ yrs. and even 75+ yrs. with little impairments and few acquired co‐morbidities do benefit from ACT in UICC stage III CC. Limitations of our analysis are the missing prospective exploration within a randomized trial design because of the nature of a registry trial. In regard to the growing amount of elderly patients, future RCTs should include and characterize elderly cancer pts. A geriatric assessment like the G‐8 screening tool should be standard of care while assessing elderly that might benefit from ACT and to establish evidenced practice guidelines.^32^

## CONFLICT OF INTEREST

Stefanie Nöpel Dünnebacke: Honoraria/Advisory Boards: BMS, Merck, and Roche. Anke Reinacher‐Schick: Honoraria: Amgen, AstraZeneca, BMS, Celgene, Lilly, Merck Serono, MSD, Pfizer, Roche, Sanofi‐Aventis, Servier, Advisory Boards: Amgen, AstraZeneca, BMS, Celgene, Lilly, Merck Serono, MSD, Pfizer, Roche, Sanofi‐Aventis, Servier, Baxalta, Pierre‐Fabre, Trial support: Amgen, AstraZeneca, Celgene, Ipsen, Lilly, Roche, Servier, and Mologen Berlin. Andrea Tannapfel: Honoraria/Advisory Board/ Trial Support: Roche, Amgen, Falk, and Celgene.

## AUTHOR CONTRIBUTION

Stefanie Noepel‐Duennebacke; substantial contributions to conception and design, analysis and interpretation of data, drafting the manuscript, and final approval of the version to be published Hendrick Jütte; analysis and interpretation of data, and final approval of the version to be published Robin Denz; analysis and interpretation of data, drafting the manuscript, and final approval of the version to be published Inke Sabine Feder; acquisition of data and final approval of the version to be published Anna‐Lena Kraeft; drafting the manuscript and final approval of the version to be published Celine Lugnier; acquisition of data and final approval of the version to be published Christian Teschendorf; acquisition of data and final approval of the version to be published Daniela Collette; acquisition of data and final approval of the version to be published Hinrich Böhner; acquisition of data and final approval of the version to be published Lars Engel; acquisition of data and final approval of the version to be published Lothar Mueller; acquisition of data and final approval of the version to be published Frank Hartmann; acquisition of data and final approval of the version to be published Ulrich Kaiser; acquisition of data and final approval of the version to be published Harald‐Robert Bruch; acquisition of data and final approval of the version to be published Stefan Hollerbach; acquisition of data and final approval of the version to be published Dirk Arnold; acquisition of data, interpretation of data, and final approval of the version to be published Nina Timmesfeld; substantial contributions to conception and design, analysis and interpretation of data, and final approval of the version to be published Andrea Tannapfel; substantial contributions to conception and design and final approval of the version to be published Iris Tischoff; substantial contributions to conception and design and final approval of the version to be published Anke Reinacher‐Schick; substantial contributions to conception and design, drafting the manuscript, and final approval of the version to be published.

## GERMAN REGISTRY OF CLINICAL TRIALS NUMBER

DRKS00004305

## ETHICAL APPROVAL STATEMENT

Local ethics committee number: 4449–12, AIO‐KRK‐0413/ass.

## Data Availability

The data that support the findings of this study are available from the corresponding author upon reasonable request.
